# Exploration of the *Muribaculaceae* Family in the Gut Microbiota: Diversity, Metabolism, and Function

**DOI:** 10.3390/nu16162660

**Published:** 2024-08-12

**Authors:** Yiqing Zhu, Borui Chen, Xinyu Zhang, Muhammad Toheed Akbar, Tong Wu, Yiyun Zhang, Li Zhi, Qun Shen

**Affiliations:** 1 College of Food Science and Nutritional Engineering, China Agricultural University, National Center of Technology Innovation (Deep Processing of Highland Barley) in Food Industry, National Engineering Research Center for Fruit and Vegetable Processing, Beijing 100083, China; yiqingzhu@163.com (Y.Z.); chenboruiabc@163.com (B.C.); xinyuzhang0319@163.com (X.Z.); toheedakbar007@gmail.com (M.T.A.); estela_tong@163.com (T.W.); 18681357759@163.com (Y.Z.); zl_litchi@163.com (L.Z.); 2 Department of Meat Science and Technology, University of Veterinary and Animal Sciences, Lahore 54000, Pakistan

**Keywords:** gut microbiota, *Muribaculaceae*, polysaccharides, short-chain fatty acids, chronic diseases

## Abstract

The gut microbiota are mainly composed of *Bacteroidetes* and *Firmicutes* and are crucial for metabolism and immunity. *Muribaculaceae* are a family of bacteria within the order *Bacteroidetes*. *Muribaculaceae* produce short-chain fatty acids via endogenous (mucin glycans) and exogenous polysaccharides (dietary fibres). The family exhibits a cross-feeding relationship with probiotics, such as *Bifidobacterium* and *Lactobacillus*. The alleviating effects of a plant-based diet on inflammatory bowel disease, obesity, and type 2 diabetes are associated with an increased abundance of *Muribaculaceae*, a potential probiotic bacterial family. This study reviews the current findings related to *Muribaculaceae* and systematically introduces their diversity, metabolism, and function. Additionally, the mechanisms of *Muribaculaceae* in the alleviation of chronic diseases and the limitations in this field of research are introduced.

## 1. Introduction

The second genome of the gut microbiota encodes 100 times more genes than the human genome [1]. These microbiota can affect the digestion and absorption of dietary components, and the resulting metabolites help the host regulate the immune system, maintain intestinal mucosal barrier function, and resist pathogens [2,3]. Previous studies have shown that plant-based diet intake is inversely correlated with obesity, hypertension, type 2 diabetes, and cardiovascular disease [4,5]. A plant-based diet is rich in active biological ingredients such as dietary fibre and polyphenols [6]. The gut microbiota can interact with these ingredients, which in turn affects the body’s nutritional metabolism and immunity [7]. Therefore, elucidating the diversity, metabolism, and function of the gut microbiota is crucial for improving human health.

The word “probiotic” comes from the Greek word “biotikos,” which means “beneficial to life”. In 2001, the Food and Agriculture Organisation of the United Nations and the World Health Organisation redefined probiotics as living microorganisms that, when consumed in sufficient quantities, can have a positive effect on the health of the host [8]. The use of probiotics depends on their source, and different probiotics have different beneficial effects on health [9]. For example, *Lactobacillus plantarum* Shinshu N-07 isolated from fermented *Brassica rapa* L. has anti-obesity effects via decreasing serum triglycerides, inhibiting hepatic steatosis, and alleviating inflammatory cell infiltration in diet-induced obese mice [10]. *Bacillus amyloliquefaciens* TL106 isolated from Tibetan pig faeces can stabilise the gut microbiota and alleviate intestinal injury induced by enterohaemorrhagic *Escherichia coli* in mice [11]. Ideally, strains used in food should be of human origin and should be able to attach to intestinal epithelial cell membranes and make active substances that benefit host metabolism without affecting the normal microbial community.

In recent years, *Muribaculaceae* have attracted much attention because of their beneficial role in maintaining host health. *Muribaculaceae* can produce short-chain fatty acids and regulate intestinal barrier function and the immune response, and they are considered a promising “next generation probiotic” [12]. Several studies have focused on genomics to reveal the gene sequence and function of the *Muribaculaceae* family [13]. To date, few isolates have been obtained from *Muribaculaceae*, and the results obtained via 16S rRNA high-throughput sequencing technology represent *norank_f_Muribaculaceae* [14]. Although few studies have investigated *Muribaculaceae*, the potential effects on host physiology have yet to be clarified. Thus, the present study systematically summarises the current research achievements related to *Muribaculaceae*. By exploring the putative functional and probiotic mechanisms, we hope to understand *Muribaculaceae* comprehensively.

## 2. Materials and Methods

We conducted a comprehensive search of research and reviews on the *Muribaculaceae* family in three databases: Google Scholar (https://scholar.google.com/), Web of Science (http://apps.webofknowledge.com), and PubMed (https://pubmed.ncbi.nlm.nih.gov/). The keywords searched included *Muribaculaceae* family, functional foods, inflammatory bowel disease, obesity, and type 2 diabetes between 2014 and 2024. To identify more relevant studies, we examined the reference lists of the studies included in the current review. On the basis of the title and abstract, we excluded some irrelevant citations and searched 105 articles for further evaluation.

## 3. Diversity of *Muribaculaceae*

The Gram-negative *Bacteroidetes* genus, including *Muribaculaceae*, *Bacteroidaceae*, *Rikenellaceae*, and *Prevotellaceae,* is one of the most abundant in the gut microbiota of mammals [1]. *Muribaculaceae* belong to Bacteroidia and Bacteroidales, which are predominantly present in the intestinal tracts of endotherms [15]. *Muribaculaceae* are highly abundant in the intestinal tract of mice, accounting for 54.99–83.44% of the total *Bacteroides* content [13]. Faeces from C57BL/6J mice constitute the primary source of *Muribaculaceae* [12], which were previously known as *Candidatus homeothermaceae* or family S24-7. In 2019, Ilias et al. reviewed the relevant data, reannotated and renamed this family *Muribaculaceae,* and used metagenomics to cluster 685 species [15]. To date, the isolates and gene sequences of this family have been identified. As shown in Table 1, 10 genera have been described in the family *Muribaculaceae*: *Muribaculum* [16], *Duncaniella* [17,18], *Paramuribaculum*, *Sodaliphilus*, *Heminiphilus* [19], *Lepagella*, *Candidatus amulumruptor*, *Candidatus merdivivens*, *Candidatus homeothermus* and *Sangeribacter*. Among these, *Muribaculum* and *Duncaniella* comprise the highest content [13].

## 4. Metabolism of *Muribaculaceae*

### 4.1. Polysaccharides

*Muribaculaceae* can metabolise endogenous and exogenous polysaccharides, including α-glucan, plant glycan, and host glycan [16]. As the dominant family of *Bacteroidetes*, *Muribaculaceae* encode a large number of enzymes that hydrolyse carbohydrates. These enzymes account for approximately 6% of the gene sequence and use starch as the basic source of energy [12]. Youn et al. used metagenomic and metatranscriptomic analyses and reported that the *Muribaculum* and *Duncaniella* genera have a high proportion of carbohydrate-metabolising genes [13]. At the species level, *Muribaculaceae* contain various enzymes that hydrolyse carbohydrates. For example, *Muribaculum gordoncarteri*, *Duncaniella dubosii*, and *Duncaniella freteri* present high activities of β-glucosidase, α-arabinase, and α-fucosidase [17,18], whereas *Heminiphilus faecis* presents α-arabinase activity and positive reactions in fermentation experiments involving mannan and L-arabinose [19]. The results of animal assays revealed that dietary fibres such as resistant starch and inulin significantly increase the abundance of *Muribaculaceae* in the gut microbiota (Table 2). Mammals do not have the enzymes to hydrolyse dietary fibre. *Muribaculaceae* can use dietary fibre as an energy source, which promote their colonisation in the gut.

The scarcity of dietary fibre in the host diet prompts *Muribaculaceae* to metabolise host glycan for usage [40]. Mucin, a highly glycosylated protein, is the primary major source of nutrients for the gut microbiota [41]. Lee et al. used Raman spectroscopy and metagenomics to analyse mucin-degrading bacteria in the mouse colon and reported that *Muribaculaceae* encode O-glycanases and sialidases [40]. O-glycanase is a critical enzyme for mucin degradation [42], whereas sialidase cleaves the terminal sialic acid and sulphate residues from mucin O-glycans [43]. Similarly, Pereira et al. used Raman-activated cell sorting, mini-metagenomics, and single-cell stable isotope probing and identified *Muribaculaceae* as major mucin monosaccharide foragers in the intestinal tract [44]. In recent years, *Akkermansia muciniphila* has been deemed a new generation of probiotics because of its ability to degrade mucin and regulate intestinal permeability and barrier integrity [45]. *Muribaculaceae* are also a mucin-degrading bacterium; however, their potential probiotic effects need to be explored further.

### 4.2. Short-Chain Fatty Acids

**Figure 1:**
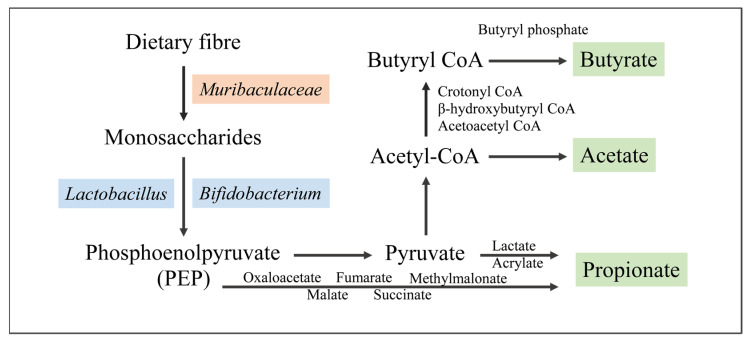
Metabolic pathways of *Muribaculaceae* to produce short-chain fatty acids.

Short-chain fatty acids are produced through the glycolysis pathway of dietary fibre in intestinal microorganisms, and the core metabolic ability of the *Muribaculaceae* family is the degradation of various complex polysaccharides [46]. Byron et al. reported that the *Muribaculaceae* family produces propionic acid [14], whereas Kate et al. reported that it produces acetic acid, propionic acid, and succinate [12]. Table 2 shows that some dietary fibre interventions, such as inulin, resistant starch, and soluble fibre, increased the abundance of the *Muribaculaceae* family in the intestinal tract of mice. Moreover, the abundances of *Muribaculum*, *Paramuribaculum*, and *Duncaniella* and the contents of acetic, propionic, and butyric acids increased markedly. In addition, potato [34] and lotus seeds [31] have high contents of resistant starch, and black cherry powder [35] is rich in pectin. A similar phenomenon was observed in animal intervention assays on these plant-based foods rich in dietary fibre. These results suggest that *Muribaculaceae* can produce short-chain fatty acids by metabolising dietary fibre (Figure 1). *Lactobacilli* intake increased the abundance of the intestinal microbiota of the *Muribaculaceae* family and short-chain fatty acids simultaneously. For example, *Lactobacillus delbrueckii* and *Streptococcus thermophilus 1131* increased the content of propionic and butyric acids [36], whereas *Lactobacillus plantarum Y44* increased the levels of acetic, propionic, butyric, and valeric acids [37]. Some studies have shown that *Lactobacillus* only encodes genes that produce lactic acid, which can be used by microorganisms that decompose polysaccharides to produce butyric acid [47]. Therefore, it can be inferred that the *Muribaculaceae* family can produce short-chain fatty acids and cross-feed with other bacteria to produce short-chain fatty acids.

### 4.3. Probiotics

Positive and negative interspecific relationships have been established between various populations of intestinal microbes, among which positive relationships include co-operation, symbiosis and cross-feeding [48]. *Bacteroidetes* are among the most abundant intestinal microbiota that interact strongly with other species. For example, *Bacteroidetes* has many outer membrane vesicles that share glycan metabolites with other microbiota [1]. The *Muribaculaceae* family is a vital member of *Bacteroidetes* that can tolerate and reduce low intestinal oxygen levels. It can adapt to and improve the intestinal environment and can coexist with other species. Table 3 shows that the ingestion of *Bifidobacterium bifidum* NK175, *Bifidobacterium lactis* XLTG11, and *Bifidobacterium longum* BR-108 increased the abundance of the *Muribaculaceae* family in the intestinal tract of mice. However, the relationship between *Muribaculaceae* and *Bifidobacterium* has rarely been reported. Previous studies have shown that *Bacteroides* and *Bifidobacterium* metabolise endogenous and exogenous glycans as symbionts [49]. Specifically, *Bifidobacterium* uses oligosaccharides, whereas *Bacteroides* breaks down polysaccharides into oligosaccharides that can be fed to *Bifidobacterium*. Since *Muribaculaceae* are active users of polysaccharides in the gut, we speculated that there is an interspecific cross-feeding relationship between *Muribaculaceae* and *Bifidobacterium*. In addition, *Lactobacillus plantarum* Shinshu N-07, *Lactobacillus paracasei* NL41, *Saccharomyces boulardii* BR14, *Streptococcus thermophilus* 1131, and *Faecalibacterium prausnitzii* significantly increased the *Muribaculaceae* content in the gut of mice (Table 3). Overall, *Muribaculaceae* are strongly correlated with probiotics, such as *Bifidobacterium* and *Lactobacillus*, and exhibit a cross-feeding or co-operative symbiotic association.

### 4.4. Others

The *Muribaculaceae* family produces vitamins for the host, including B1 (thiamine), B2 (riboflavin), B3 (niacin), B5 (pantothenic acid), B7 (biotin), and B9 (folate). This phenomenon could be attributed to the ability of the *Muribaculaceae* family to encode genes encoding vitamin transporters [12]. In terms of amino acid metabolism, metagenomics predicted that 75–100% of the species in the *Muribaculaceae* family synthesise aspartate, glutamine, glutamic acid, glycine, methionine, valine, leucine, and isoleucine [13]. In addition, *Muribaculaceae* also produce some rare enzymes in *Bacteroidetes*, such as genes encoding ureases, IgA-degrading peptidases, and oxalate-degrading enzymes [40]. Importantly, the *Muribaculaceae* family produces oxaloyl CoA decarboxylase and formyl CoA transferase for the degradation of oxalic acid [60]. Most plant-based foods contain oxalic acid, which might underscore the plant-based diet-promoted growth of *Muribaculaceae* in the gut.

## 5. Functions of *Muribaculaceae*

### 5.1. Inflammatory Bowel Disease (IBD)

A complete mucus barrier is the first line of defence that protects intestinal health, and impaired mucus barrier function is one of the major signs of IBD [61]. The structure and function of mucus depend mainly on mucin, which moistens and lubricates the intestinal wall. These properties protect intestinal epithelial cells from mechanical stress and exert immune effects, enhancing intestinal homeostasis [43]. The *Muribaculaceae* family is attached to the mucous layer and is a symbiotic user of myxoglycan in the intestine, showing a strong correlation with IBD [40]. Strikingly, IBD decreases the abundance of the *Muribaculaceae* family in the intestinal microbiota. Volk et al. reported that the abundance of the *Muribaculaceae* family was significantly lower in the mucous layer of *Nlrp6*^−/−^ and *IL18*^−/−^ deficient mice than in an intact mucous layer [62]. A similar phenomenon has been observed in animal models of IBD, such as following the administration of dextran sulphate sodium (DSS), lipopolysaccharide (LPS), and excessive antibiotics [63,64], because IBD destroys the structural integrity of the mucous layer, which is the attachment site and a major nutrient source of the *Muribaculaceae* family [65]. Some plant foods (ginger and cranberry bean) and plant-derived active substances, such as dietary fibre (garlic and fucoidan polysaccharides), polyphenols (*Sophora flavescens* extract, oryzanol), oligosaccharides (galactose oligosaccharide and 2′-fucosyllactose), saponins (*Pulsatilla* saponin), and terpenoids (total diterpenoids), alleviate the symptoms of IBD (Table 4). Concurrently, these interventions increase the abundance of *Muribaculaceae* and improve intestinal barrier function. The role of *Muribaculaceae* in IBD remission may be affected via the following three mechanisms: (1) Increased gene expression levels and regeneration of mucin alleviate colon tissue injury and reduce intestinal permeability [41]. (2) The metabolism of polysaccharides produces short-chain fatty acids that stimulate the release of mucus and activate the signalling pathway to exert anti-inflammatory effects [66]. (3) The species competes with pathogens for the ecological niche and nutrients in the intestinal mucus layer and resists the colonisation of intestinal pathogens [44]. In summary, IBD destroys the mucous layer of the intestinal tract of mice, decreasing the number of ecological sites of intestinal symbiotic bacteria and the abundance of *Muribaculaceae*. Consequently, the expression of mucin decreases, which damages the mucous layer, resulting in a vicious cycle. Effective intervention substances for alleviating IBD are beneficial for the colonisation of *Muribaculaceae* and the recovery of the mucus layer.

### 5.2. Type 2 Diabetes (T2D)

T2D is a metabolic disorder characterised by hyperglycaemia and insulin resistance [79]. The gut microbiota are involved in the progression of T2D via host energy homeostasis, glucose and lipid metabolism, insulin sensitivity, and the inflammatory response [80]. The intake of plant-derived dietary fibres, such as *Sargassum*, Konjac glucomannan, wakame polysaccharide, moutan cortex polysaccharide, and black seed polysaccharide, alleviates insulin resistance, glucose tolerance, dyslipidaemia, and liver and kidney damage in diabetic animal models; together, these features are related to an increased presence of the *Muribaculaceae* family in the intestinal microbiota (Table 5). Notably, polysaccharides from *Sargassum*, wakame polysaccharides, and black seed polysaccharides increase the abundance of *Muribaculaceae* and activated the insulin receptor/phosphatidylinositol-3-kinase/protein kinase B (IRS/PI3K/Akt) signalling pathway. These phenomena regulate the insulin signal transduction and glucose metabolism pathways, in turn increasing glucose uptake and glycogen synthesis [6,81]. Mannan plays a synergistic role with metformin in the treatment of C57BL/6J diabetic mice; this therapeutic effect is related to the increased abundance of *Akkermansia muciniphila* in the *Muribaculaceae* family [82]. Additionally, acarbose treats diabetes by preventing the breakdown of starch in the small intestine and altering the composition of the gut microbiota. Byron et al. used metagenomics and reported that the nine most responsive bacteria under the intervention of acarbose are classified in the *Muribaculaceae* family [14]. Although further investigation is needed on other bacterial genera/species that improve blood glucose metabolism and insulin resistance, these findings suggest that an increase in *Muribaculaceae* could help exert the anti-diabetic properties of hypoglycaemic drugs.

### 5.3. Obesity

Obesity underlies the disruption of the intestinal microbiota, and an increase in the *Firmicutes*/*Bacteroides* ratio is considered one of the indicators of obesity [89]. The *Muribaculaceae* family is a major member of *Bacteroides*, and a significant negative correlation has been established between *Muribaculaceae* and the risk of obesity (Table 6). A population investigation revealed a high content of the *Muribaculaceae* family in the gut of the Yanomami people, who consumed a plant-based diet dominated by fruits and grains [90]. Osborne et al. analysed the intestinal microbiota of 248 subjects in Bangladesh and reported that the *Muribaculaceae* family was negatively correlated with body weight, hip circumference, waist circumference, and other physical indicators [91]. In a dietary survey of 29 Chinese people, Qian et al. reported a lower content of *Muribaculaceae* in the intestines of people with a high-fat diet and a higher content in those with a diet dominated by plant foods [92]. Similarly, *Muribaculaceae* abundance significantly decreased in a high-fat-diet-fed mouse model. Some functional components of plant sources, such as dietary fibre (resistant starch and asparagus soluble fibre), polyphenols (naringin), and pomegranate acid, improve the symptoms of obesity and reverse the intestinal microbiota disturbance caused by a high-fat diet, increasing the abundance of *Muribaculaceae* (Table 6). Moreover, the *Muribaculaceae* family is also involved in anti-inflammatory reactions in the gut. For example, high-amylose resistant starch increases serum adiponectin and decreases the level of the inflammatory factor IL-17; these phenomena are related to increased *Muribaculaceae* family content [93]. Muhomah et al. reported that the decreased level of secretory immunoglobulin A in mice fed a high-fat diet is related to reduced *Muribaculaceae*, acetic acid, and butyric acid contents [94]. Together, these studies reveal the relevant role of the gut microbiota in regulating metabolic disorders and immune inflammation, suggesting that probiotics, especially *Muribaculaceae*, could be used as a therapeutic tool for obesity.

## 6. Conclusions

In summary, we conducted a comprehensive review of studies related to the *Muribaculaceae* family to find evidence of associations between *Muribaculaceae* and plant foods, functional components, and various diseases. The results showed that *Muribaculaceae* had a strong capacity to metabolise endogenous (mucin glycans) and exogenous (dietary fibre) polysaccharides, could produce short-chain fatty acids, and had cross-feeding relationships with *Bifidobacterium* and *Lactobacillus*. Additionally, IBD, obesity, and T2D cause intestinal microbiota imbalance and decrease the abundance of the *Muribaculaceae* family. The intake of plant-derived foods (cereals, fruits, and tea), plant-derived active functional components (polysaccharides, polyphenols, and saponins), and probiotics (*Lactobacillus*, *Bifidobacterium,* and yeast) is conducive to the intestinal colonisation of the *Muribaculaceae* family. However, several issues related to the *Muribaculaceae* family need to be investigated: (1) The functional identification of *Muribaculaceae* isolates has been based mainly on mouse faeces, whereas that in the human gut has rarely been studied. (2) The reasons for the decline in *Muribaculaceae* in disease models of IBD, obesity, and T2D, as well as the molecular mechanisms of disease alleviation, remain to be explored. (3) The cross-feeding relationship between *Muribaculaceae* and probiotics such as *Bifidobacterium* and *Lactobacillus* needs to be substantiated by in vitro fermentation culture.

## Figures and Tables

**Table 1:** Diversity of *Muribaculaceae* family.

Bacteria Genus	Strain	Name	Genome Assembly	GenBank	References
*Muribaculum*	*Muribaculum gordoncarteri*	TLL-A4	ASM480369v1	GCA_004803695.1	Miyake et al., 2020 [16]
	*Muribaculum intestinale*	YL27	ASM168884v2	GCA_001688845.2	Lagkouvardos et al., 2016 [20]
	*Muribaculum* sp. *An287*, *Muribaculum* sp. *An289*	An287, An 289	ASM215958v1	GCA_002159585.1	Schoch et al., 2020 [21]
*Duncaniella*	*Duncaniella dubosii*, *Duncaniella freteri*	H5, TLL-A3	ASM480391v1, ASM476612v1	GCF_004803915.1, GCF_004766125.1	Miyake et al., 2020 [18]
	*Duncaniella muris*	DSM 103720	ASM302480v1	GCA_003024805.1	Lagkouvardos et al., 2019 [15]
*Paramuribaculum*	*Paramuribaculum intestinale*	DSM 100764	ASM302481v1	GCA_003024815.1	Lagkouvardos et al., 2019 [15]
*Sodaliphilus*	*Sodaliphilus pleomorphus*	Oil-RF-744-WCA-WT-10	ASM967695v1	GCA_009676955.1	Wylensek et al., 2020 [22]
*Heminiphilus*	*Heminiphilus faecis*	AM35T	ASM872896v1	GCA_008728965.1	Schoch et al., 2020 [21]
*Lepagella*	*Lepagella muris*	NM04_E33	ASM479397v1	GCA_004793975.1	Afrizal et al., 2022 [23]
*Candidatus amulumruptor*	*Candidatus amulumruptor caecigallinarius*	Hinsu1	ASM2074201v1	GCA_020742015.1	Hinsu et al., 2019 [24]
*Candidatus merdivivens*	*Candidatus merdivivens faecigallinarum*, *Candidatus merdivivens pullicola*, *Candidatus merdivivens pullistercoris*	B3-2255	ASM1769505v1, ASM1769493v1	GCA_017695055.1, GCA_017694935.1	Gilroy et al., 2021 [25]
*Candidatus homeothermus*	*Candidatus homeothermus arabinoxylanisolvens*	M4	/	/	Ormerod et al., 2016 [12]
*Sangeribacter*	*Sangeribacter muris*	A43	/	/	Forster et al., 2021 [26]

**Table 2:** Intake of polysaccharides, plant foods, and probiotics increases *Muribculaceae* abundance and short-chain fatty acid content.

Intervention Substance	Subject	*Muribaculaceae* Abundance	Short-Chain Fatty Acid	References
Inulin	Dairy cows	↑	Lactic acid, propionic acid, butyric acid	Wang et al., 2021 [27]
Soluble fibre	C57BL/6J mice	↑	Acetic acid, propionic acid	Xu et al., 2020 [28]
Resistant starch	C57BL/6 mice	↑	Acetic acid, propionic acid, butyric acid	Wan et al., 2021 [29]
Inulin	CD-1 mice	↑	Acetic acid, propionic acid, butyric acid	Zou et al., 2024 [30]
Resistant starch	BALB/c mice	↑	Acetic acid, butyric acid	Li et al., 2023 [31]
Corderan gum	C57BL/6J mice	↑	Acetic acid, propionic acid, butyric acid	Watanabe et al., 2021 [32]
Konjac glucomannans	SD rat	↑	Butyric acid	Deng et al., 2023 [33]
Potatoes	SD rat	↑	Acetic acid, propionic acid, butyric acid	Wu et al., 2019 [34]
Black cherry powder	SD rat	↑	Acetic acid, propionic acid, butyric acid	Garcia-Mazcorro et al., 2018 [35]
*Lactobacillus delbrueckii*, *Streptococcus thermophilus* 1131	ICR mice	↑	Propionic acid, butyric acid	Usui et al., 2018 [36]
*Lactobacillus plantarum* Y44	C57BL/6J mice	↑	Acetic acid, propionic acid, butyric acid, valerate acid	Liu et al., 2020 [37]
*Lacticaseibacillus casei* ATCC393	BALB/c mice	↑	Lactic acid, acetic acid	Aindelis et al., 2021 [38]
*Lactobacillus acidophilus, Bacillus subtilis*	Piglets	↑	Butyric acid	Xie et al., 2022 [39]

The up arrow indicates an increase in abundance.

**Table 3:** The intake of probiotics increases the abundance of *Muribaculaceae*.

Intervention Substance	Subject	*Muribaculaceae* Abundance	References
*Lactobacillus plantarum* Shinshu N-07, *Lactobacillus curvatus* #4G2	C57BL/6J mice	↑	Yin et al., 2020 [10]
*Lactobacillus kefirnofaciens* M1, *Lactobacillus mali* APS1	C57BL/6J mice	↑	Lin et al., 2020 [9]
*Lactobacillus paracasei* NL41	SD rat	↑	Zeng et al., 2021 [50]
*Lactobacillus plantarum* NK151, *Bifidobacterium bifidum* NK175	C57BL/6 mice	↑	Yun et al., 2021 [51]
*Lactobacillus plantarum* Y44	BALB/c mice	↑	Gao et al., 2021 [52]
*Bifidobacterium longum* BR-108	BALB/c mice	↑	Makioka et al., 2018 [8]
*Bifidobacterium lactis* XLTG11, *Lactobacillus casei* Zhang, *Lactobacillus plantarum* CCFM8661, *Lactobacillus rhamnosus* Probio-M9	BALB/c mice	↑	Li et al., 2023 [53]
*Saccharomyces boulardii* BR14	C57BL/6J mice	↑	Mu et al., 2021 [54]
*Saccharomyces boulardii*	C57BL/6J mice	↑	Dong et al., 2019 [55]
*Bacillus amyloliquefaciens* TL106	C57BL/6J mice	↑	Bao et al., 2021 [11]
*Lactobacillus plantarum*, *Weissella confusa*	C57BL/6 mice	↑	Gryaznova et al., 2024 [56]
*Faecalibacterium prausnitzii*	BALB/c mice	↑	Hu et al., 2021 [57]
*Weissella confuse*, *Pediococcus acidilactici*, *Ligilactobacillus equi*	KM mice	↑	Pei et al., 2021 [58]
*Lactobacillus plantarum* QP28-1, *Bacillus subtilis* QB8	Bamei piglets	↑	Zhang et al., 2024 [59]

The up arrow indicates an increase in abundance.

**Table 4:** An increase in *Muribaculaceae* is related to the alleviation of IBD.

Intervention Substance	Subject	*Muribaculaceae* Abundance		References
		Changes before Intervention	Changes after Intervention	
Infliximab and adalimumab	IBD patients	↓	/	Alatawi et al., 2021 [67]
Recombinant mouse Il18	C57BL/6 mice	↓	/	Volk et al., 2019 [62]
N-acetylcysteine	C57BL/6J mice	↓	↑	Wang et al., 2021 [61]
Cranberry beans	C57BL/6 mice	↓	↑	Monk et al., 2016 [68]
Flavones from matrine	C57BL/6 mice	↓	↑	Shao et al., 2021 [69]
Garlic polysaccharide	C57BL/6J mice	↓	↑	Shao et al., 2020 [70]
Oryzanol	C57BL/6J mice	↓	↑	Xia et al., 2022 [71]
2′-Fucosyllactose	C57BL/6J mice	↓	↑	Li et al., 2020 [63]
Cucurbitacin E	C57BL/6J mice	↓	↑	Zhan et al., 2024 [72]
Cinnamon essential oil	KM mice	↓	↑	Li et al., 2020 [65]
Fresh ginger	BALB/c mice	↓	↑	Guo et al., 2021 [73]
Butyrate	BALB/c mice	↓	↑	Kang et al., 2023 [74]
Pulsatilla saponin	SD rat	↓	↑	Liu et al., 2021 [75]
Fucoidan	C57BL/6J mice	↓	↑	Luo et al., 2021 [64]
Euphorbia total diterpenoids	C57BL/6J mice	↓	↑	Wang et al., 2021 [76]
Galactooligosaccharide	Piglet	↓	↑	Gao et al., 2021 [77]
Rosmarinic acid	ICR mice	↓	↑	Wang et al., 2023 [78]

The up and down arrows indicate increases and decreases in abundance.

**Table 5:** An increase in *Muribaculaceae* content is related to the alleviation of T2D.

Intervention Substance	Subject	*Muribaculaceae* Abundance		References
		Changes before Intervention	Changes after Intervention	
Acarbose	C57BL/6J mice	/	↑	Smith et al., 2021 [14]
Metformin, saxagliptin, and repaglinide	C57BL/6J mice	↓	↑	Tang et al., 2024 [83]
Black seed polysaccharide	KM mice	/	↑	Dong et al., 2020 [84]
Loquat leaf sesquiterpene	db/db mice	/	↑	Chen et al., 2021 [85]
Broad-spectrum antibiotics	db/db mice	↓	/	Yu et al., 2019 [86]
Gegen Qinlian decoction	db/db mice	/	↑	Liu et al., 2024 [79]
Konjac glucomannan	SD rat	↓	↑	Deng et al., 2020 [87]
Sargassum polysaccharide and acarbose	SD rat	↓	↑	Li et al., 2021 [6]
Wakame polysaccharide	SD rat	↓	↑	Li et al., 2021 [81]
Moutan cortex polysaccharide	SD rat	↓	↑	Zhang et al., 2022 [88]
Mannooligosaccharides	C57BL/6J mice	↓	↑	Zheng et al., 2021 [82]
*Morus alba* L. water extract	C57BL/6J mice	↓	↑	Du et al., 2022 [80]

The up and down arrows indicate increases and decreases in abundance.

**Table 6:** An increase in *Muribaculaceae* is related to alleviating obesity.

Intervention Substance	Subject	*Muribaculaceae* Abundance		References
		Changes before Intervention	Changes after Intervention	
Olive oil, lard oil, soybean oil	C57BL/6J mice	/	↓	Liu et al., 2021 [95]
Trans-fatty acids	C57BL/6J mice	/	↓	Hua et al., 2020 [96]
10% alcohol solution	C57BL/6J mice	/	↓	Júnior et al., 2019 [97]
Pu-erh tea extract	C57BL/6J mice	↓	↑	Ye et al., 2021 [98]
Resistant starch	C57BL/6J mice	↓	↑	Barouei et al., 2017 [93]
Naringin	C57BL/6J mice	↓	↑	Mu et al., 2020 [99]
Jabuticaba peel	C57BL/6J mice	↓	↑	Loubet et al., 2022 [100]
Saskatoon berry	C57BL/6J mice	↓	↑	Zhao et al., 2023 [5]
Prebiotic oligofructose	C57BL/6J mice	↓	↑	Paone et al., 2023 [101]
Chlorogenic acid	C57BL/6J mice	↓	↑	Yu et al., 2024 [102]
Punicic acid	ICR mice	↓	↑	Yuan et al., 2020 [103]
Fu brick tea	KM mice	↓	↑	Zhou et al., 2020 [104]
Asparagus soluble fibre	KM mice	↓	↑	Zhang et al., 2021 [105]

The up and down arrows indicate increases and decreases in abundance.
